# Time-Frequency Analysis of Motor Imagery During Plantar and Dorsal Flexion Movements Using a Low-Cost Ankle Exoskeleton

**DOI:** 10.3390/s25102987

**Published:** 2025-05-09

**Authors:** Cristina Polo-Hortigüela, Mario Ortiz, Paula Soriano-Segura, Eduardo Iáñez, José M. Azorín

**Affiliations:** 1 Brain-Machine Interface Systems Lab, Miguel Hernández University of Elche, 03202 Elche, Spain; cpolo@umh.es (C.P.-H.); mortiz@umh.es (M.O.); p.soriano@umh.es (P.S.-S.); jm.azorin@umh.es (J.M.A.); 2 Engineering Research Institute of Elche—I3E, Miguel Hernández University of Elche, 03202 Elche, Spain; 3 Valencian Graduated School and Research Network of Artificial Intelligence—ValGRAI, 46022 Valencia, Spain

**Keywords:** electroencephalography (EEG), brain–machine interface (BMI), time-frequency transforms, motor imagery, low-cost exoskeleton, neurorehabilitation, inertial measurement units (IMUs)

## Abstract

Sensor technology plays a fundamental role in neuro-motor rehabilitation by enabling precise movement analysis and control. This study explores the integration of brain–machine interfaces (BMIs) and wearable sensors to enhance motor recovery in individuals with neuro-motor impairments. Specifically, different time-frequency transforms are evaluated to analyze the correlation between electroencephalographic (EEG) activity and ankle position, measured by using inertial measurement units (IMUs). A low-cost ankle exoskeleton was designed to conduct the experimental trials. Six subjects performed plantar and dorsal flexion movements while the EEG and IMU signals were recorded. The correlation between brain activity and foot kinematics was analyzed using the Short-Time Fourier Transform (STFT), Stockwell (ST), Hilbert–Huang (HHT), and Chirplet (CT) methods. The 8–20 Hz frequency band exhibited the highest correlation values. For motor imagery classification, the STFT achieved the highest accuracy (92.9%) using an EEGNet-based classifier and a state-machine approach. This study presents a dual approach: the analysis of EEG-movement correlation in different cognitive states, and the systematic comparison of four time-frequency transforms for both correlation and classification performance. The results support the potential of combining EEG and IMU data for BMI applications and highlight the importance of cognitive state in motion analysis for accessible neurorehabilitation technologies.

## 1. Introduction

Stroke and spinal cord injuries represent a significant contributor to the loss of motor function in individuals. In addition to the motor sequel of the patient, two critical factors must be considered. The first is the exponential increase in the number of patients affected. The second is linked to the high costs of rehabilitation therapies with these kind of devices. Currently, the combination of BMIs with exoskeletons is not widely used outside of the research field. Recent studies have demonstrated that the use of this technique is associated with promising results thanks to the improvement in neuroplasticity [1,2].

A BMI is a system that processes the electroencephalographic (EEG) signals derived from brain activity in order to generate commands to control an external device. One of the most common paradigms that has been demonstrated to be effective in several studies is motor imagery [3,4]. This mental task consists of imagining the movement of a joint without executing it. For this reason, it is necessary to define optimal classification algorithms for this mental task in order to control the starting and stopping of the device.

Signal processing constitutes a crucial component of such algorithms. Recent studies have highlighted the importance of adequate signal processing in motor imagery classification due to the inherently noisy and non-stationary nature of EEG signals. Appropriate preprocessing and feature extraction steps are essential to improving signal quality and isolating relevant cognitive patterns. For example, ref. [5] showed that standardization and feature selection not only improve classification accuracy, but also significantly reduce training time. In a large-scale deep learning study, ref. [6] found that models trained on raw EEG performed poorly compared with those trained on minimally preprocessed data, especially for MI tasks. Furthermore, ref. [7] highlighted the critical role of artifact removal in preserving discriminative features within EEG signals. A variety of signal processing methods are currently available for the purpose of extracting features. Some of the most common paradigms used are common spatial patterns (CSPs) [8] and Power Spectral Density (PSD) [9]. Less common is the use of frequency-time transforms. These transforms allow the combined analysis of the behavior of a signal in both the time and frequency domains. In this study, three of them are used: the Stockwell transform (ST), the Chirplet transform (CT) and the Hilbert–Huang transform (HHT). In addition, their performance is evaluated in comparison with the Short-Time Fourier transform (STFT), which is applied to short time windows to analyze how the frequency content of non-stationary signals evolves over time. The main advantage of using time-frequency transforms over traditional methods is their ability to achieve better temporal and spectral resolution and to characterize local oscillatory patterns in the signal. This is particularly relevant for understanding the dynamic and non-stationary nature of EEG signals, especially in populations or contexts with high inter-individual variability [10]. In addition, these transforms allow for the more accurate detection of transient events and rapid changes in the signal. Another important advantage is their ability to minimize noise and artifacts in the signal [11,12]. The Chirplet transform, for example, has been shown to be effective in improving the classification of P300 potentials in BCI systems [13] and has also been applied in EEG analysis for seizure detection [14]. Although to a smaller extent, some recent work has explored its usefulness in decoding motor imagery tasks [15]. In the case of the Stockwell transform, its effectiveness has been demonstrated in studies aimed at localizing transient events, such as in the detection of epileptic activity [16] or in the analysis of EEG during the gait cycle [17]. This transform has also been used in the design of algorithms for BCI [18]. It has also been used in combination with deep learning techniques or feature selection strategies to improve the performance of classification models [19,20]. Finally, the Hilbert–Huang transform (HHT) has been applied in a variety of contexts, such as real-time detection of drowsy states [21], as well as in the classification of motor imagery tasks, where it effectively captures dynamic EEG changes [22,23].

In addition to these applications, recent studies have provided evidence that time-frequency features extracted from EEG signals can also be used to decode specific kinematic parameters related to movement, thereby strengthening their relevance in BMI systems. For example, it has been demonstrated that direction and distance in reaching tasks can be reliably decoded from pre-movement EEG activity using spectral features derived from event-related desynchronization patterns [24]. Similarly, hand movement speed has been successfully estimated from EEG during drawing tasks [25], and adaptive methods have been proposed for estimating hand trajectories based on mu and beta band features extracted by the Short-Time Fourier Transform [26].

While these studies focus on decoding motor intention from EEG, another complementary approach explored in this research involves analyzing the correlation between EEG activity and actual limb movement. The purpose of obtaining such a parameter is that it could become a metric for evaluating the patient’s performance in each therapy. This would allow continuous monitoring of the patient’s condition and personalized methodologies for each patient. This individualization could be based on the choice of exercise type, intensity and duration. It is known that the cerebral potential of the legs is located in the dorsal aspect and in the medial wall of the hemisphere of the motor cortex [27]. The problem is that it is difficult to discern the laterality of the movement, which is not the case for upper-limb movement. A previous study [28] in the literature proposes a promising classification of dorsiflexion and plantar flexion movements using a combination of EEG and functional magnetic resonance imaging (fMRI). The study demonstrates that the characteristics of the Movement-Related Cortical Potential (MRCP) in the delta band across a broad spectrum can effectively discriminate between four levels of Rate of Force Development (RFD) during ankle dorsiflexion, with promising applications in brain–machine interface (BMI) technologies and neurorehabilitation. Another study [29] demonstrates that the Movement-Related Cortical Potential (MRCP) characteristics in the delta band and a broad spectrum can effectively discriminate between four levels of RFD.

As mentioned at the beginning of this section, another state-of-the-art element in neurorehabilitation therapies is the use of exoskeletons. These devices assist in the movement of specific joints and, as mentioned above, help to improve brain plasticity in patients [30,31]. However, the high cost of these devices represents an economic barrier to their accessibility [32]. This study focuses on analyzing brain activity associated with two ankle movements: dorsiflexion and plantar flexion. These movements, which play a crucial role in human gait [33], will be performed by a specially designed ankle exoskeleton.

This work has multiple purposes. Firstly, it aims to assess whether time-frequency transforms are suitable methods for estimating the correlation between electroencephalographic (EEG) signals and the position of the foot during ankle movements. Secondly, we analyze how this correlation varies with the subject cognitive involvement, comparing the states of motor imagery and relax. Thirdly, the effectiveness of these transforms in decoding motor imagery will be investigated using a deep learning-based classifier. Finally, performance and feasibility of a low-cost ankle exoskeleton specifically designed for experimental tests performed will be evaluated.

To address these objectives, experimental tests were conducted using a self-designed low-cost ankle exoskeleton. The protocol involved the performance of mental tasks (relax and motor imagery) specifically designed for future implementation in a state-machine-based control system that alternates between two modes of operation: static and motion. Four time frequency analysis methods were employed to process the EEG signals: Short-Time Fourier Transform (STFT), Stockwell Transform (ST), Hilbert–Huang Transform (HHT), and Chirplet Transform (CT). In the first part of the study, the correlation between EEG signals and foot position was evaluated across different frequency bands and under both cognitive conditions. The frequency band exhibiting the highest correlation was then selected as the basis for the second part of the study, which focused on decoding motor imagery. In this phase, the EEG signals were processed using the same four time frequency methods to classify motor imagery versus relax, independently for both the static and motion models of the exoskeleton.

This article is structured in several sections. Section 2 describes both the elements used in the study (participants, equipment and experimental protocol) and the different stages of preprocessing and processing of the EEG signals. This is followed by Section 3, which details the two main studies conducted in this research. Section 4 presents the findings obtained in both studies, together with their corresponding statistical analysis. Subsequently, a discussion of the results is presented in Section 5, followed by the Section 6 dedicated to the limitations encountered during the development of the work. Finally, Section 7 summarizes the main contributions of the study and raises possible lines of future research.

## 2. Materials and Methods

This section will describe the materials used in the experiments carried out in this research as well as the methods applied in the processing of EEG signals.

### 2.1. Materials

#### 2.1.1. Subjects

A total of six subjects (S1, S2, S3, S4, S5 and S6) participated in this experiment. None of the subjects exhibited any diagnosed motor or neural dysfunction. The ages of the subjects ranged from 23 to 32 years old (26±3). It is important to mention that all subjects have right-lateral dominance, as the exoskeleton has been placed on their dominant leg. Prior to the experiment, participants were informed about the nature of the study and provided written consent in accordance with the Helsinki declaration. All the procedures carried out were approved by the Office of Responsible Research of the Miguel Hernández University of Elche (Spain) with reference DIS.JAP.01.22. In addition, to assess participants’ kinesthetic motor imagery capacity, all participants completed the form Movement Imagery Questionnaire-3 (MIQ-3) in its Spanish version [34]. The MIQ-3 asks participants to imagine both kinesthetically and visually the performance of simple body movements (e.g., bending a knee or lifting an arm) and then rate how difficult they found the task. By focusing on this kinesthetic dimension, the questionnaire ensures participants can maintain and perceive imagined movements, which is crucial for the reliability of the motor imagery tasks in the present study.

#### 2.1.2. Equipment

**Figure 1:**
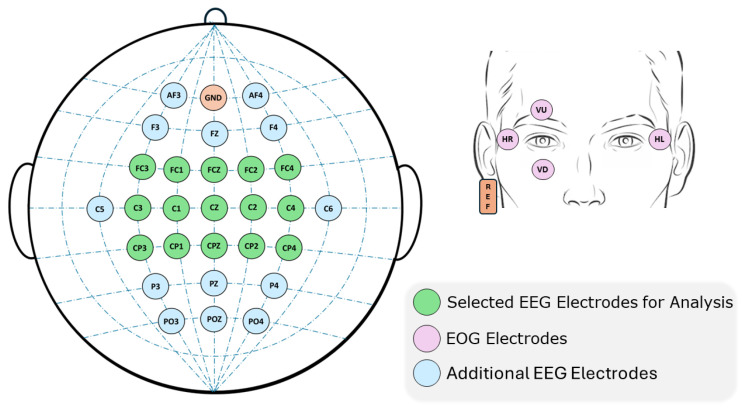
On the left side of the image, the distribution of the electrodes utilized in this research can be observed. The electrodes utilized for signal processing are indicated by a green highlight. Additional electrodes used for EEG signal acquisition are highlighted in blue. The image on the right illustrates the distribution of the EOG electrodes on the faces of the subjects. Two electrodes (VU, VD) are positioned vertically between the eyes to capture blinks. The remaining electrodes (HR, HL) are positioned laterally between the two eyes to capture horizontal movements. On the right ear, a reference (highlighted in orange) is positioned as a clamp.

The materials used in this experiment are divided into hardware and software components. The first hardware component is the brain signal recording device: g.NAUTILUS PRO FLEXIBLE from g.tec medical engineering GmbH, Austria [35]. This system has a set of g.SCARABEO electrodes connected to a Wi-Fi transmitter. The signals from this transmitter are picked up by a base station which is connected via USB to the computer. The sampling frequency is selectable between 250 and 500 Hz. This cap features a total of 32 non-invasive wet electrodes and 3 accelerometers. Of the total number of electrodes, 28 are used based on the 10-10 distribution of the international system: AF3, F3, FZ, FC3, FC1, FCZ, C5, C3, C1, CZ, CP3, CP1, CPZ, P3, PZ, PO3, AF4, F4, FC2, FC4, C2, C4, C6, CP2, CP4, P4, POZ, and PO4. The AFZ electrode serves as the common ground (GND) and the reference electrode is placed on the subject’s right earlobe. The remaining four electrodes Visual Up (VU), Visual Down (VD), Horizontal Left (HL), Horizontal Right (HR) are utilized for collecting electro-oculographic (EOG) signals, which record eye movements and blinks. See Figure 1 for more detail.

**Figure 2:**
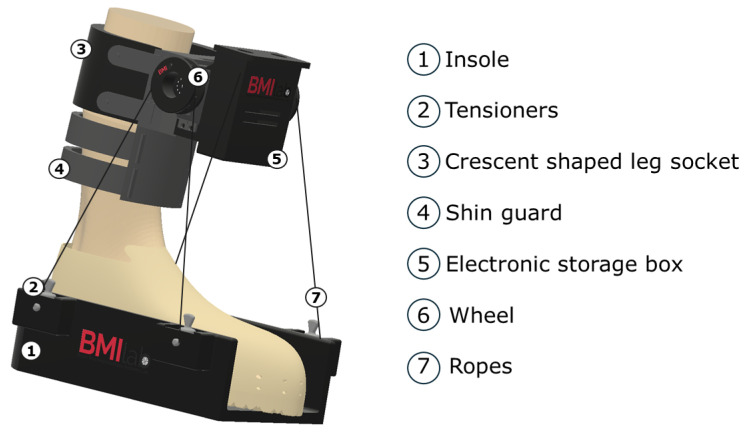
Three-dimensional design of the developed ankle exoskeleton. The modular design with its three main elements is easily distinguishable.

An essential component of the equipment used is the low-cost ankle exoskeleton developed, as shown in Figure 2. It has been designed with a modular philosophy that facilitates dorsiflexion and plantar flexion movements. At the bottom of the device, there is a footplate on which the subject places the foot. This insole has four grip points: two at the front and two at the back.

The second part of the device is positioned over the subject’s shin and consists of a crescent-shaped piece that connects to a kind of shin guard using a ratchet closure. This element is the grey-colored piece that can be seen again in Figure 2. The electronic storage box of the device is attached to this latter part via a T-shaped union. In this section, two servo motors are located, each at one end. Each servo motor is equipped with two wheels, which are connected via four ropes to the four grip points on the insole. A dorsal flexion movement is generated when the movement of the wheels lifts the front part of the insole. Conversely, when the movement of the wheels creates tension on the back of the insole, it forces the subject to perform a plantar flexion.

As can be seen, the device has been designed with a low-cost philosophy, both in terms of the structural design, based on 3D printing, and the use of simple, low-cost electronic components. The total cost of the exoskeleton, including all mechanical and electronic parts, is approximately EUR 100.

**Figure 3:**
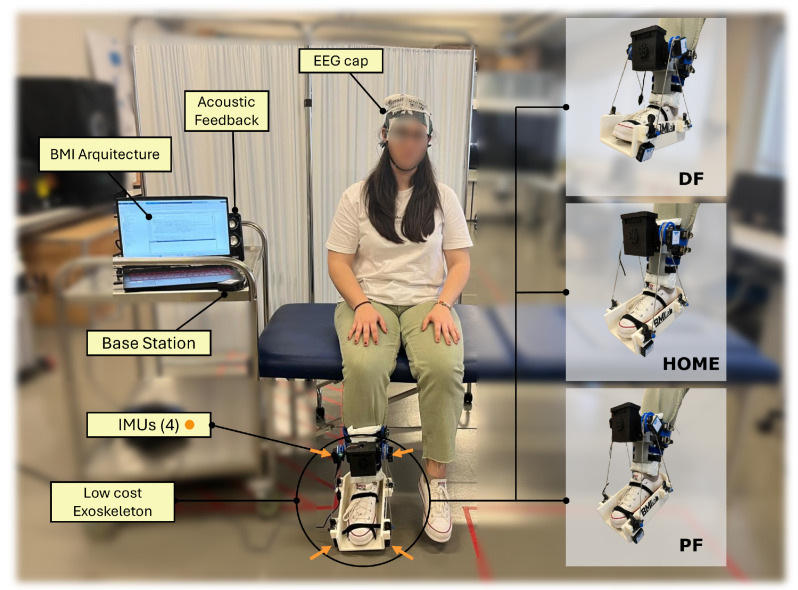
The following image shows the arrangement of the subject and the materials used in one of the experiments. The computer with the developed BMI architecture is located on the left side. In addition, the rest of the elements such as the EEG cap data reception base and the loudspeakers for the auditory indicators are shown. In the middle of the picture, the subject is seated on a stretcher. The EEG cap and the EOG electrodes are located on the head. The ankle exoskeleton is located on the user’s dominant leg. The left-hand side shows the three positions of the exoskeleton. It starts from an initial position (home) to perform a dorsal flexion. It then returns to the home position to initiate a plantar flexion. When this movement is finished, it returns to the home position. All this is performed cyclically until it receives the order to stop.

Another hardware component used in this research provides the position of the exoskeleton. For this purpose, four IMUs (Inertial Measurement Units) of WIT Motion company are used [36]. In order to have better feedback of the subject’s foot movement, an IMU is positioned on each wheel and two are placed at the right and left sides of the insole (see Figure 3). The IMUS work at a sampling frequency of 100 Hz, so a subsequent resampling is necessary to be able to work together with the EEG signal.

A final component used is a set of loudspeakers to indicate the mental task the user must perform. The complete setup used in the tests and the arrangement of the subjects in the experiment can be seen in Figure 3.

The software used for data acquisition, preprocessing, and EEG signal processing is based on a BMI architecture developed in MATLAB R2022b by the research group.

#### 2.1.3. Experimental Protocol

One of the main objectives of this research is to design an optimal control algorithm to enable future real-time closed-loop control. At this stage, model performance is explored in open-loop, although the ultimate goal remains a closed-loop system. However, as the ultimate purpose is the closed-loop, the protocol is designed with that aim. For this purpose, the protocol is based on sustained motor imagery and a state machine. The state machine manages the transition from a *static* model, which will be used for starting the exoskeleton, to a *motion* model, which will be used for stopping the exoskeleton, in a similar way to previous investigations [37].

**Figure 4:**
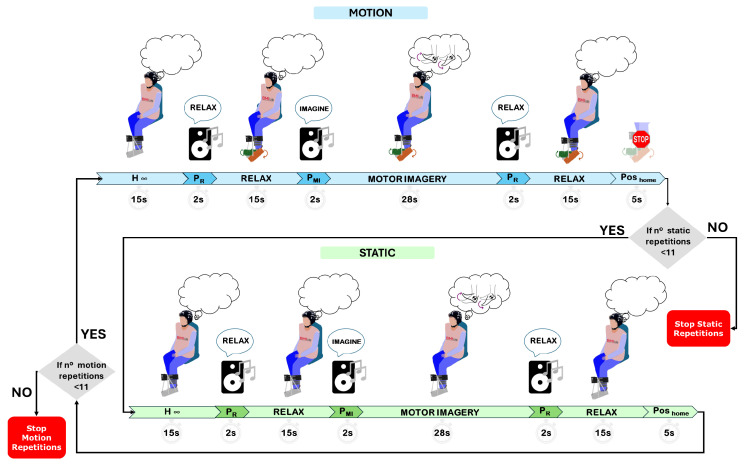
This image shows the protocol followed in the open-loop tests of this research. The blue color shows the order and duration of motion trials. The same but in green for the static trials. The only difference between the two types of trials is that after the H Infinity period, the exoskeleton performs cyclic movements of PF and DF only in motion trials. Motion and static trials alternate until 11 of each are completed. All mental tasks are preceded by an auditory cue in the form of a voice.

Two types of trials corresponding to these models are performed: the first one with the exoskeleton, which is completely immobile (static), and the second featuring continuous dorsiflexion (DF) and plantar flexion (PF) (motion). These movements can be seen in Figure 3. In total, 22 trials are performed, alternating between 11 motion trials and 11 static trials; see Figure 4. During each trial, the subject must complete two mental tasks. The first, named *Relax*, requires the subject to maintain a state of mental relaxation. The second, called *Motor Imagery*, requires the subject to perform a kinesthetic motor imagination of the foot on which the exoskeleton is located.

The experimental protocol is illustrated in Figure 4. All trials begin with a 15-s period to allow the ocular filter to converge. Next, an acoustic signal indicates the start of the Relax task, which has a duration of 15 s. After this period, another audio signal marks the start of the Motor Imagination task, which runs for 28 s. Finally, a third auditory signal indicates the start of a second Relax task, with the same duration as the first one. At the end, an additional 5 s are added in which the exoskeleton, in the movement tests, is ordered to return to the initial position, allowing the subject to adopt a comfortable posture at the end of the test.

### 2.2. Methods

**Figure 5:**
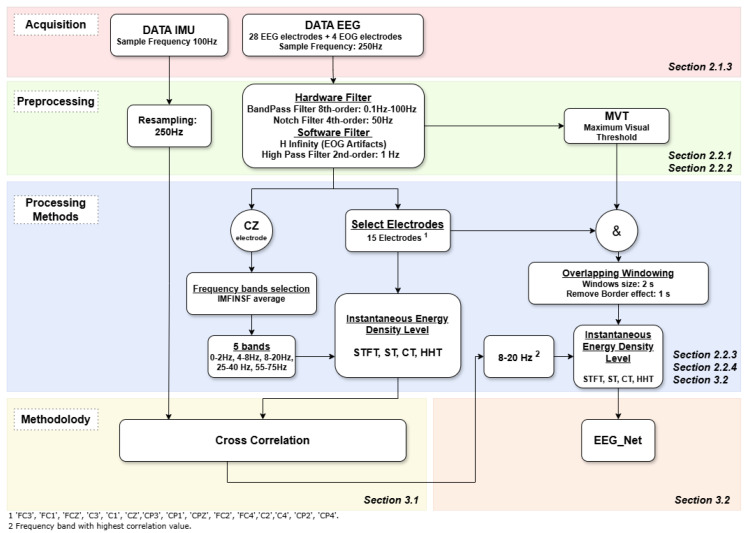
This figure describes a general outline of the working process of this research. The study starts with the acquisition of data by performing six experimental tests. After that, the signal is de-artifacted by using hardware filters and software. For signal processing, the different frequency-time transforms are used. The purpose of all this is to obtain the Instantaneous Energy Density Level. Prior to this, a standardization of the frequency bands to be used is carried out. Finally, the two lines of research are proposed. On the one hand, we study the analysis of the correlation between the position of the foot and the EEG signal. On the other hand, we perform decoding of the motor imagination. For the first study, only the motion model data as well as the unnormalized data are used. For the second study, the normalized data as well as the data from both the motion and static trials are used.

Figure 5 summarizes the whole process followed in this research. This section concerns the first four steps corresponding mainly to the signal pre-processing and processing methods employed. The first part of this section describes the pre-processing and filtering of the signal. Then, the signal processing techniques in the time and frequency domain are detailed.

#### 2.2.1. Signal Preprocessing

This preliminary step in the analysis of EEG signals is essential to remove information that is not relevant for signal decoding. EEG and EOG signals are sampled at a frequency of 250 Hz. Two types of hardware and software filters are applied. First, an 8th-order Butterworth hardware bandpass filter with a passband of 0.1 to 100 Hz is applied. Second, a 4th-order Butterworth hardware notch filter with a center frequency of 50 Hz is used to remove interference from the Spanish power grid.

The subsequent processing steps involve software filters designed to mitigate ocular artifacts, including flicker and horizontal movements. This is achieved using an H-infinity filter [38]. This filter treats each electrode as an independent subsystem by dynamically adjusting its gain in real time, minimizing the worst-case impact of unmodelled disturbances and thereby effectively suppressing ocular artifacts, baseline drifts, and recording biases. Once the electro-oculography (EOG) signal has been filtered, a low-frequency component remains, which may be undesirable for analysis. Therefore, a 2nd-order Butterworth software high-pass filter with a cutoff frequency of 1 Hz, implemented in state-space form, is applied individually to each EEG channel.

A selection of electrodes (Figure 1) is made to reduce computational costs and to focus on the areas related to motor control. This choice of electrodes covers the premotor area, the supplementary motor area and the primary motor cortex [39]. The fifteen electrodes selected are as follows: ‘FC3’, ‘FC1’, ‘FCZ’, ‘C3’, ‘C1’, ‘CZ’, ‘CP3’, ‘CP1’, ‘CPZ’, ‘FC2’, ‘FC4’, ‘C2’, ‘C4’, ‘CP2’, ‘CP4’.

#### 2.2.2. Normalize Data for Motor Imagery Decoding Analysis

A final pre-processing step is the normalization of the signal. For this purpose, the MVT (Maximum Visual Threshold) method is used in a similar way to other studies [18]. It is important to note that this process is applicable only to motor imagery analysis. For each channel, MVT was estimated by averaging the six highest amplitude values of the signal. This calculation was performed for each time window shift, and the results were stored in a vector. The MVT for each window was updated using the average of this vector. For the first epoch of the initial training trial, the BMI used the MVT thresholds from the generic file of model 0, based on data from a previous subject. This information was updated for each epoch, converging to a stable value after several seconds, ensuring it does not affect the analyzed events. Subsequent trials use the updated thresholds from the previous trials. The normalization of the signal follows this equation:

(1) $$SV{\left(t\right)}_{ch}=\frac{V{\left(t\right)}_{ch}}{\frac{1}{Ch}\sum_{j=1}^{Ch}MVT_{j}}$$

where Equation (1), Ch is the total number of electrodes, $V{\left(t\right)}_{ch}$ is the value of the signal in channel ch at time *t*, and $MVT_{j}$ is the maximum visual threshold value for channel *j*.

#### 2.2.3. Data Analysis

This section describes the four methods that will be used to process the EEG signal. These methods will be applied in different ways depending on the two studies proposed in this article. On the one hand, we will use the common processing technique based on the Fourier Transform [40]. This methodology will be compared with other types of techniques that allow us to know the instantaneous frequency and amplitude, the latter being the one we will be working with. The frequency-time transforms used will be Hilbert–Huang Transform (HHT) [41], Stockwell Transform (ST) [42] and Chirplet Transform (CT) [43]. All the techniques used will be employed to obtain the instantaneous energy density level of the signal per frequency band.

##### Short-Time Fourier Transform

The Short Time Fourier Transform (STFT) decomposes a time signal into short and overlapping segments by applying the Fourier Transform to each segment as Equation (2) indicates. This allows an analysis of how frequencies vary as a function of time, which is useful for non-stationary signals.

(2) $$X\left(m,k\right)=\sum_{n=-\infty }^{\infty }x\left[n\right]\cdot w\left[n-mR\right]\cdot e^{-j\frac{2\pi kn}{N}}$$

In the same way as in the previous methods, the final objective is to know the variation of the energy over time. Therefore, we proceed in the same way to integrate the time-frequency signal composition obtained in Equation (3) in the same way as defined for ST.

(3) $$IE{\left(t\right)}_{stft}=\int_{w_{i}}{\left|X(m,k)\right|}^{2}\cdot dk$$

##### Stockwell Transform

The Stockwell transform (ST) is designed to provide a time-frequency representation of a signal [42]. Its operation is based on a scaled localizing Gaussian window (4).

(4) $$S\left(\tau ,f\right)=\int_{-\infty }^{\infty }x\left(t\right)\frac{\left|f\right|}{\sqrt{2\pi }}e^{-\frac{{\left(\tau -t\right)}^{2}f^{2}}{2}}e^{-j2\pi ft}\;$$

This transform allows us to know the energy value of the signal as a function of frequency and time (5).

(5) $$E\left(\tau ,f\right)={\left|S(\tau ,f)\right|}^{2}$$

As we are working with the magnitude of the energy over time, we must integrate this energy along the second dimension of the resulting matrix (6). This will result in the instantaneous energy density level for a time t.

(6) $$IE{\left(t\right)}_{stockwell}=\int_{f_{i}}E\left(\tau ,f\right)\cdot df$$

##### Hilbert–Huang Transform with VMD

Hilbert–Huang Transform (HHT) combines two techniques: a decomposition and the Hilbert transform [44]. Instead of the traditional Empirical Mode Decomposition (EMD), this study uses Variational Mode Decomposition (VMD) algorithm. VMD is a signal processing technique that decomposes a signal into a set of Intrinsic Modal Functions (IMFs), which are well defined in terms of time and frequency. This method achieves this decomposition by iterating several key steps including Fourier transforms, center frequency updates, and the application of Lagrange multipliers. VMD differs from EMD in that it uses a variational approach that minimizes the bandwidth of the modes in the Fourier domain, leading to more robust and stable decompositions. VMD’s optimization framework provides a well-defined mathematical basis, enhancing its robustness to noise and improving the quality of mode separation compared to the heuristic and recursive nature of EMD [45].

The first step of this method is to transform the original signal x(t) into the time domain by means of the Fourier transform $\hat{x}\left(\omega \right)$. Each IMF is defined in the frequency domain as Equation (7) where λ(ω) is the *k* in the frequency domain, λ(ω) is the Lagrange multiplier in the frequency domain and α is the penalty factor.

(7) $${\hat{u}}_{k}\left(\omega \right)=\frac{\hat{x}\left(\omega \right)-\sum_{i\neq k}{\hat{u}}_{i}\left(\omega \right)+\frac{\lambda (\omega )}{2}}{1+\alpha {\left(\omega -\omega_{k}\right)}^{2}}$$

The middle frequencies are updated in each iteration as:

(8) $$\omega_{k}=\frac{\int_{0}^{\infty }\omega {\left|{\hat{u}}_{k}\left(\omega \right)\right|}^{2}\;d\omega }{\int_{0}^{\infty }{\left|{\hat{u}}_{k}\left(\omega \right)\right|}^{2}\;d\omega }\approx \frac{\sum_{\omega }\omega {\left|{\hat{u}}_{k}\left(\omega \right)\right|}^{2}}{\sum_{\omega }{\left|{\hat{u}}_{k}\left(\omega \right)\right|}^{2}}$$

The Lagrange multiplier is updated at each iteration as follows, where τ is the update rate.

(9) $$\lambda \left(\omega \right)=\lambda \left(\omega \right)+\tau \left(\hat{x}\left(\omega \right)-\sum_{k}{\hat{u}}_{k}\left(\omega \right)\right)$$

Finally, each IMF is transformed into the temporal domain by Inverse Fourier Transform.

(10) $$u_{k}\left(t\right)=F^{-1}\left\{{\hat{u}}_{k}\left(\omega \right)\right\}$$

Once the value of the IMF is obtained, the Hilbert Transform (HT) (11) is applied. HT allows us to calculate both the amplitude (12) and the instantaneous frequency (13).

(11) $${\hat{u}}_{k}\left(t\right)=H\left\{u_{k}\left(t\right)\right\}=\frac{1}{\pi }P.V.\int_{-\infty }^{\infty }\frac{u_{k}\left(\tau \right)}{t-\tau }d\tau$$

(12) $$z_{k}\left(t\right)=u_{k}\left(t\right)+i{\hat{u}}_{k}\left(t\right)=a_{k}\left(t\right)e^{i\theta_{k}\left(t\right)}$$

(13) $$\omega_{k}\left(t\right)=\frac{d\theta_{k}\left(t\right)}{dt}$$

In addition to these results, it also allows us to calculate the value of the Instantaneous Energy $E_{k}\left(t\right)$ as Equation (14). This magnitude will be the final variable that will be used in this research when the HHT method is mentioned. The instantaneous energy will be calculated for each value of IMF. With this value, it will be possible to know the variation of the energy signal over time.

(14) $$E_{k_{\mathrm{hht}}}\left(t\right)={\left|z_{k}\left(t\right)\right|}^{2}=u_{k}{\left(t\right)}^{2}+{\hat{u}}_{k}{\left(t\right)}^{2}$$

##### Chirplet Transform

The Chirplet Transform (CT) is a method that allows signals with non-stationary frequency components to be analyzed [46]. Unlike traditional transforms, which assume that the frequency of signals is constant over time, the Chirplet Transform can handle signals whose frequency changes continuously. This is achieved by introducing a frequency modulation rate known as “chirp”.

(15) $$C\left(\tau ,f\right)=\int_{-\infty }^{\infty }x\left(t\right)g\left(t-\tau \right)e^{-j2\pi ft}e^{-j\pi \alpha {\left(t-\tau \right)}^{2}}\;dt$$

Equation (15) represents the mathematical formula of the Chirplet transform, where the $e^{-j\pi \alpha {\left(t-\tau \right)}^{2}}$ is the quadratic frequency modulation term referred to as chirp. In the same way as Fourier Transform and Stockwell Transform, it is necessary to integrate the result of the transform. Therefore, after this transformation, the variation of the energy over time is obtained.

(16) $$IE{\left(t\right)}_{chirplet}=\int_{f_{i}}{\left|C(\tau ,f)\right|}^{2}\cdot df$$

#### 2.2.4. Frequency Bands Selection

As different methods are used to study the time-frequency signal, it is necessary to standardize the selection of the frequency bands. For this purpose, the Hilbert–Huang transform will be used as a reference.

The HHT is known for its ability to decompose non-stationary and non-linear signals into IMFs, which represent oscillatory components with varying instantaneous frequencies. These instantaneous frequency values are obtained after applying the Hilbert transform to each IMF (Section 2.2.3). By averaging the IMFs’ instantaneous frequencies (IMFINSF), a representative measure of the frequency characteristics of each IMF is obtained, allowing an adaptive and accurate selection of frequency bands.

(17) $$\mathrm{IMFINSF}\left(t\right)=\frac{1}{2\pi }\frac{d}{dt}\arg\left(z_{k}\left(t\right)\right)$$

An iterative process was used to calculate the averaged frequency of each IMF. For all the subjects and trials, the average IMFINSF value of each IMF for the CZ electrode was calculated for each mental task period into which the signal was divided (first relaxation, motor imagery and second relaxation). The reason for using this electrode is that it is closest to where the brain activity related to lower-limb movement originates [47]. Then, for each subject, a global mean was taken of these frequency values obtained for each IMF and for each mental task. This gives five mean values corresponding to the five IMFs obtained from the iterative process of the Hilbert–Huang transform. Finally, these five frequencies are averaged across the six subjects in order to determine the mean value of each of the frequency bands associated with each IMF. The results of these calculations for each model can be seen in Table 1. Then, they are extended to characterize the five frequency bands which could be related to traditional EEG rhythms [48]: 0–2 Hz (delta), 4–8 Hz (theta), 8–20 Hz (alpha, low beta and mid beta), 25–40 Hz (high beta) and 55–75 Hz (gamma).

#### 2.2.5. Statistical Analysis

To ensure the robustness and reliability of the findings, statistical analyses were performed on all studies and results presented in this research. The purpose of these analyses was to determine whether the observed differences between methods, frequency bands, and subject performance were statistically significant. All analyses were carried out using IBM SPSS Statistics 24 software [49].

For the first analysis, statistical evaluation was performed to assess whether the correlation values between EEG signals and ankle position differed significantly depending on the frequency band examined, the time-frequency transformation applied (STFT, HHT, ST, CT), and the mental task performed (relax vs. motor imagery).

The second statistical analysis, related to the decoding of motor imagery, was conducted separately for the motion and static conditions. The primary aim was to determine whether the classification performance of the different time-frequency transform methods (STFT, HHT, ST, and CT) differed significantly. In addition, the analysis examined whether decoding accuracy varied according to the subject and the mental task class (relax vs. motor imagery).

## 3. Methodology

As shown in Figure 5, this section describes the two studies proposed in this research. On the one hand, Section 3.1 develops the procedure used to see if there is a certain correlation between the variation of the energy of the signal over time and the position of the exoskeleton. On the other hand, the analysis of the signal to decode motor imagery is explained in Section 3.2 in order to find the optimal control algorithm for the exoskeleton.

### 3.1. Correlation Analysis of Motion Trials

One of the main objectives of this article is to determine which frequency bands have a higher correlation between the position of the exoskeleton and the EEG signal. This implies that only motion trials are used for this analysis. The electrode selection for this initial study mirrors the one presented in Section 2.2.1. For each trial and electrode, the instantaneous energy is computed across each of the five defined frequency bands. Subsequently, these energy values are averaged to obtain the mean energy across all trials for each electrode and frequency band. This process is repeated for the four time-frequency transforms to be applied in this study, as well as for the three signal segments per mental task into which the signal has been divided (Relax, MI, Relax).

From this, correlation between the exoskeleton signal (from an IMU) and the average electrode energy in each segment is calculated using the cross-correlation method. This technique allows the calculation of the cross-correlation (XCF) between two time series with different lags [50]. In this study, a progressive smoothing approach was used to ensure comparability across different frequency bands. Signal smoothing was implemented using a multi-level moving average filter, with each level of smoothing corresponding to a variable-size window. These smoothing windows were calculated based on the IMU period and sampling frequency to ensure proper temporal alignment between the signals. The purpose of the smoothing was to normalize the variability across frequency bands, allowing a clearer and a more accurate comparison with the IMU signal. Cross-correlation was used to identify the optimum delay between the smoothed signal and the IMU signal by assessing the similarity at different times. This approach made it possible to determine how the smoothed signal aligns with the IMU signal at different levels of smoothness, providing a more detailed understanding of their temporal relationship and facilitating the detection of coherent patterns between the signals.

**Figure 6:**
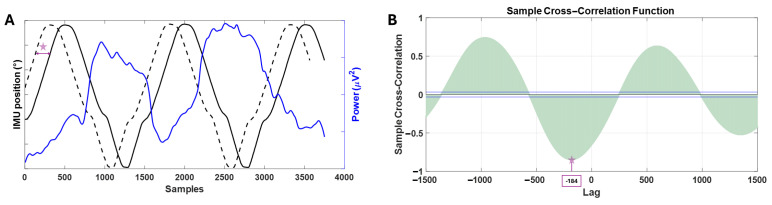
Three signals can be seen in (**A**). The blue signal is the variation of the instantaneous power in a section of the first relaxation for one electrode and for one subject. The black continuous signal is the position marked by the IMU of the foot position. The black dashed signal is the signal ahead of the same lag value where the maximum correlation value has been given. The star represents the delay between both signals. In (**B**), look at the correlation values between the two signals for different lags. The blue lines represent the statistical significance threshold for the cross–correlation function. In this example, the maximum correlation value is observed for a lag of −184 samples (indicated with the star), which is the same as the one shifted in (**A**). It is noticeable that there is some negative correlation between the EEG signal and the IMU signal.

Three signals can be seen in Figure 6A. The blue signal represents the instantaneous energy variation for a section of the test. The black continuous signal illustrates the position of the exoskeleton during the same section. The black dashed signal represents the IMU signal advanced the same number of samples corresponding to the phase shift where the maximum correlation value is found. Figure 6B shows the cross-correlation result at different lags.

In order to obtain a global metric of the correlation values for the fifteen selected electrodes, the absolute average is calculated per subject, per frequency band, and per segment. This analysis will differentiate the correlation behavior based on the relaxation and motor imagery segments as well as the five studied frequency bands. The frequency band that shows the highest average value among the four time-frequency transforms employed will be used to calculate the variation of instantaneous energy in the feature extraction described in Section 3.2.

To determine the average lag values where the highest correlation values occur, an average is calculated, and the values are averaged for the three segments in each band. This final step will provide the average lags for each frequency band.

### 3.2. Motor Imagery Decoding

The objective of this study is to define the signal processing methodology for feature extraction. Given that the application of this algorithm must be carried out in real time, the overlapping windowing technique is employed. This analysis involves dividing the signal by windows or epochs of a size of 2 s, with a window overlap of 1.5 s. This results in signal predictions every 0.5 s.

The feature vector will be determined by the variation of the instantaneous energy in each window. This process will be repeated for the four methods defined in Section 2.2.3. The accuracy of each method will be evaluated individually and for the two models that would be employed in the state machine: motion and static models. In the case of feature extraction for the frequency-time transforms (HHT, ST and CT), the border effects they generate have to be taken into account. The border effects generated by such small processing windows are detrimental, as they alter the signal’s behavior. Consequently, in the two-second analysis window, 0.5 s are removed from each border. Finally, only the central one-second segment is selected as a feature per epoch.

All epochs are labelled with the mental task that the subject is executing in each trial. For model training, epochs corresponding to task transitions are removed.

In summary, our algorithm will be programmed to process the Instantaneous energy variation of a two-second window. Every 0.5 s, it will be able to predict whether the subject is engaged in motor imagery (class 1) or, on the contrary, is in a relaxed state (class 0).

This research makes use of deep learning algorithms by employing the EEGNet neural network [51]. This network is specifically designed for EEG signal classification and uses depth convolutions and separable convolutions to optimize spatial and temporal feature extraction. Depth convolutions learn spatial filters within temporal convolutions, reducing the number of parameters, while separable convolutions combine these spatial filters across temporal bands. The network contains adjustable parameters such as number of classes, channels, time points, dropout rate and dropout type. For this research, the values of the parameters are as follows: number of classes = 2, channels = 15, epochs = 110, dropoutRate = 0.35, batch size = 128, epochs = 600.

The evaluation method of this algorithm will be based on a leave-one-out cross-validation. A total of 22 models will be generated per subject, 11 static and 11 dynamic models. Ten trials of each type will be used to build and train the model. The remaining trial of each model, not used for training, will be used to validate the model as a test trial. During training, the Adam optimizer is used with an adaptive learning rate controlled by a cosine learning rate scheduler known as Cosine Annealing Scheduler [52]. This method dynamically adjusts the learning rate between a maximum and a minimum value during training cycles, helping to improve model convergence and avoid falling into local minima. In addition, callbacks are used to store the best model based on the F1 metric, which balances accuracy and recall, and to adjust the learning rate. After training, the best model is selected based on its performance on the validation set. This selected model is used to make predictions for each epoch of the test trial. Thus, each 2-s epoch of the test trial is preprocessed, processed and classified as a 0 (relax) or 1 (Motor Imagery) in a period of time lower than the 0.5 s shifting in order to simulate the real-time behavior of the BMI.

## 4. Results

This section presents the results of the two studies described in the previous section. Firstly, the correlation values for each frequency band and for each mental task are evaluated. Secondly, the performance of the proposed motor imagery decoding algorithm is evaluated.

### 4.1. Motion Correlation Results

In this section, the results of the signal correlation analysis are presented, organized into key aspects that highlight the most significant findings.

- Average correlation per band and mental task.

**Figure 7:**
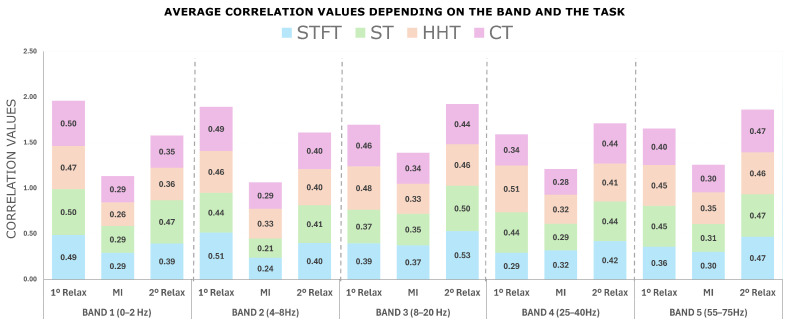
This figure shows the average correlation values of the 15 electrodes obtained for each frequency band and for each mental task section. In blue, data corresponding to STFT, in green to ST, in orange to HHT and in pink to CT. It can be seen that the band with the greatest difference between the relaxation and motor imagination sections is the band corresponding to 4–8 Hz. It is also noticeable that the band with the best correlation values is the 8–20 Hz band, with STFT and HHT having the highest average values.

The results obtained from the correlation analysis, detailed in Section 3.1, are presented in Figure 7. As can be seen in this figure, these results are categorized based on the five frequency bands studied and the three main segments involved in the trials. The correlation values have been normalized to a range between 0 and 1, as the average of the absolute values was taken.

Three critical aspects can be identified, as observed in Figure 7. First, there is a significant difference between the correlation values for the relaxation and motor imagery segments across all transformations. The frequency band that shows the greatest difference is 4–8 Hz for all transformations, except for Hilbert–Huang, which shows a greater difference in the 0–2 Hz band. The ST and STFT demonstrate the most prominent differences.

Secondly, when evaluating which frequency band presents the highest correlation value for the relaxation and motor imagery segments, the results clearly indicate that the 8–20 Hz band is the most prominent. Therefore, this band will be used for feature extraction as mentioned in Section 3.2. The transformations that show the highest correlation values in this band are HHT and STFT.

- Correlation per electrode

**Figure 8:**
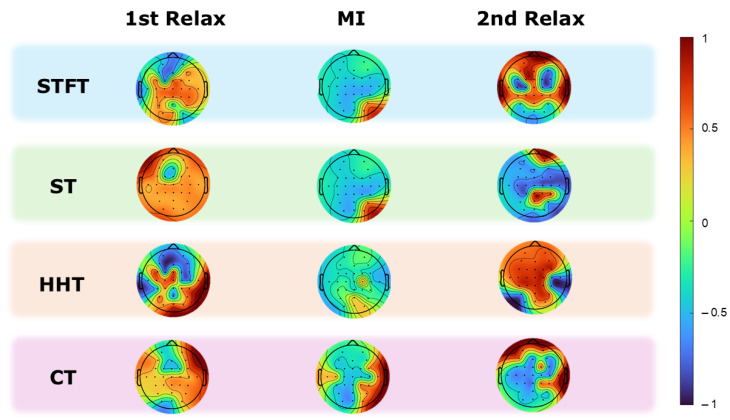
This figure is divided horizontally by the three frequency-time methods used in this research. It is also divided vertically by the three mental tasks performed in the experiments. The topoplots show the correlation values obtained for all electrodes for subject S2 and for the frequency band 8–20 Hz. It is clearly distinguishable how there is a change in the value of the correlations between the relaxation sections, whose values tend to increase, and between the motor imagination section, which tends to decrease.

Figure 8 illustrates the correlation behavior for a subject across the four different transformations and three distinct segments in the 8–20 Hz band. For HHT, a high correlation is observed in the first relaxation at the selected electrodes, as previously discussed in Section 2.2.1. A decrease is also observed during the MI task, with the greatest reduction observed in the central and frontocentral electrodes. In contrast, a partial increase is observed in the second relaxation. The STFT also shows a similar pattern, with high correlation in the first relaxation, a marked reduction during MI, particularly at the central electrodes, and a partial recovery in the second relaxation. The CT data exhibit a high correlation in the central and parietal regions during the first relaxation, a decrease in the central region during MI, and a recovery in the second relaxation. In contrast, the ST data show a high correlation in the central and parietal areas during the first relaxation and a decrease during MI. Finally, an increase in correlation values is observed in the second relaxation, especially in the central and frontocentral areas.

- Differences between mental tasks

Alternatively, an examination of the values obtained for the first and second relaxation phases, as illustrated in Figure 7, reveals a notable difference. It is evident that the correlation is consistently higher for all transformations during the initial relaxation segment compared to the subsequent one.

- Differences between transforms

A general analysis of the transformation correlations across all bands reveals that the HHT transformation exhibits the highest average values.

- Lag analysis

**Figure 9:**
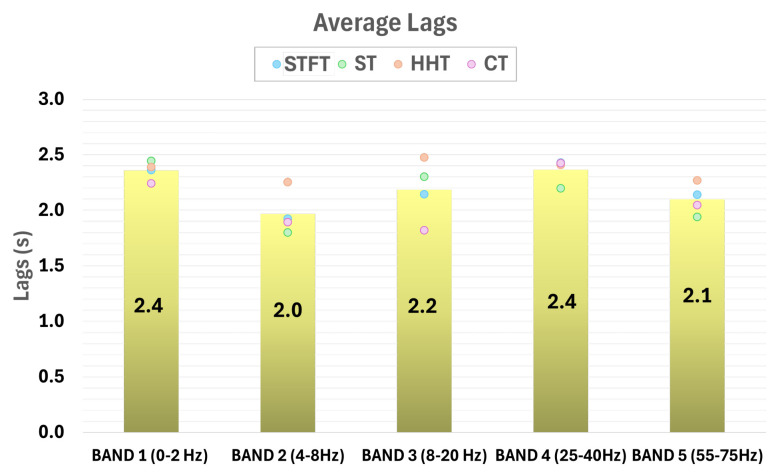
This figure shows the average lag values for each frequency band. These values for all frequency bands are around 2 s. It is noticeable that the frequency band with the lowest lag value is the 4–8 Hz band. This band was the same band where the correlation differences between MI and Relax were the highest.

One last aspect to consider in these results is the lag values where the maximum correlations were reached. It is observed that the mean lag value for all frequency bands is around 2 s, as can be seen in Figure 9. Therefore, EEG signals show its highest correlation with the IMUs’ signals when they are shifted around two seconds.

#### Statistical Analysis of Correlation Results

In order to statistically evaluate these results and to make the conclusions more robust, a statistical analysis of the results will be carried out.

The first step is to verify that the data for each method are normally distributed. This analysis is carried out using the Kolmogorov–Smirnov method, since the number of samples is greater than 50. The results of this study clearly show that for the four methods, as well as for each band and for each class, the data follow a normal distribution. This means that the study is based on parametric methods. It should be noted that no outliers were detected.

To start with a MANOVA analysis, it is necessary to check whether the assumption of sphericity is fulfilled in order to give the results more validity, as well as to apply the necessary correction factors. For this purpose, the Mauchly test is performed. This test consists of verifying if the variances of the differences between all possible pairs of conditions are equal. The result in this study is that the assumption of sphericity is violated ((p=0.017)<0.05). Sphericity is therefore rejected and the most powerful correction factor is applied, which in this case is the Huynh–Feldt correction factor. The study of the interaction between the methods leads to the conclusion that there are significant differences between them, since (p<0.05). On the other hand, if analyzing the interaction between the methods and the classes, we can see that there is a significant difference: $F_{\left(6,225\right)}=2.312,\;\eta^{2}=0.058,\;p<0.05$. This means that the performance of the methods varies depending on the classes. This means that there is a significant difference between the values of the relax classes and the MI class.

### 4.2. Motor Imagery Decoding Results

This section presents the results obtained from the analysis of the motion and static trials for all subjects. The study focuses only on the variation of instantaneous energy in the 8−20 Hz band. This is because this band exhibited the highest correlation values between the EEG signals and the IMU position. In addition, it contains the alpha and part of the beta rhythms, which are the ones reported in the literature as more relevant during motor imagery tasks [53].

The results, which distinguish the accuracy rates for the mental tasks of relaxation and motor imagery, are displayed in Table 2. This table also allows for an assessment of the effectiveness of each applied transformation.

#### Statistical Analysis of Motor Imagery Decoding Results

Statistical analysis is then performed again to validate the results. The first part of this study begins with the evaluation of the results obtained for the *motion* model. In a first descriptive analysis, the final average values for each method are obtained: STFT = 93.84%, HHT = 73.50%, ST = 89.28% and CT = 91.55%. As can be seen, the average results between the STFT, CH and ST methods are very similar to each other, so a statistic analysis is needed for significance. To decide if a parametric or non-parametric test should be applied in the analysis of the methods, a normality study is carried out for each methodology. The Kolmogorov–Smirnov method is used, which is more specific for the sample size used in this study (132 samples per method). By the use of this first method, nine outliers have been detected: three of them from STFT, five from ST trials and the last one from CT. These elements have been extracted from statistical analysis. The acquired results are as follows for STFT (D(124)=0.177, p<0.001), ST (D(124)=0.173, p<0.001) and CT (D(124)=0.162, p<0.001). It can be seen that the significance values are less than 0.05, indicating that these data do not follow a normal distribution. In the case of HHT (D(132) = 0.068, *p* = 0.200), the significance value is slightly higher than 0.05, suggesting that this data set may follow a normal distribution, although not conclusively. Because most methods do not follow a normal distribution, it was decided to proceed with non-parametric tests for statistical analysis. The first test performed is Friedman’s test [54] for significant differences between the methods. This methodology revealed a statistically significant discrepancy in the average ranks of the methods (STFT, HHT, ST and CT) ($\chi^{2}$(3) = 158.823, p<0.001). This indicates that at least one of the methods differs significantly from the others. The mean ranks were 3.33 for STFT, 1.38 for HHT, 2.52 for ST and 2.77 for CT. A Wilcoxon test [55] is then performed on a pairwise basis to find out which methods differ significantly from each other and which differ to a lesser degree. This analysis indicates that STFT stands out as the most distinct and potentially superior method. ST and CT are the most similar methods to each other. To assess the differences between the categories of the variables *Class* and *Subject* in each of the methods, the Kruskal–Wallis [56] test was used. The results obtained for the subject group analysis indicate that the STFT method (p=0.700) is stable and robust among subjects. This is not the case for the other methods: HHT (p=0.001), ST (p=0.006), and CT (p=0.001), which depend significantly on the subject. In the class-oriented analysis, all methods indicate that there is no significant difference between MI and Relax: STFT (p=0.877), HHT (p=0.488), ST (p=0.663), and CT (p=0.215).

After performing the statistical analysis for the tests carried out on the motion model, the *static* model is analyzed. The averaged accuracies obtained for each transform in this case are 91.96% for STFT, 68.97% for HHT, 88.42% for ST and 89.79% for CT. In this case, the number of outliers are five: two of STFT and ST and one of CT. Regarding normality, in this case, none of the four methods follows a normal distribution: STFT (D(127)=0.175, p<0.001), ST (D(127)=0.140, p<0.001), CT (D(127)=0.177, p<0.001) and HHT (D(127)=0.093, p<0.009). Given this non-normal distribution behavior, a non-parametric analysis is performed again. The Friedman test reveals that the methods also show statistical differences between them (STFT, HHT, ST and CT) ($\chi^{2}$(3) = 204.132, p<0.001). Following this, we proceed to perform a Wilcoxon test to find out which methods are statistically more similar or different. Focusing on the differences between the STFT method and the rest, this method shows higher values compared to the rest of the methods. However, the differences are more significant between STFT and HHT. On the other hand, the differences between CT and ST are not significant, with a p<0.118). Finally, a Kruskal–Wallis study is carried out to find out if there are differences in the methods depending on the classes and subjects, as in the motion model. All methods show significant variability in their performance across different groups of subjects: STFT (p=0.001), HHT (p=0.001), ST (p=0.002), and CT (p=0.001). This suggests that the performance of each method is not uniform and has a high dependency on the subject. Finally, in the class analysis, only the HHT method exhibits different behavior depending on the class (p=0.006) while the other methods show an uniform performance for the MI and Relax classes: STFT (p=0.331), ST (p=0.686), and CT (p=0.865)

## 5. Discussion

This study (summarized in Figure 5) has investigated the relationship between EEG signals and ankle position during plantar and dorsal flexion movements in stretching with and without mental involvement. On the other hand, the decoding of motor imagery was evaluated in order to define a state machine for the development of an exoskeleton control algorithm. All these analyses were carried out by evaluating the performance of two types of methods: the short-time Fourier transform (STFT) and the frequency-time transforms (ST, HHT and CT). The results obtained allow us to address several key aspects in neuro-motor rehabilitation and in the development of accessible technologies for this purpose.

In an effort to evaluate the correlation between EEG signals and foot position during two mental tasks—one involving mental engagement (MI) and the other not (relax)—two significant conclusions were drawn. The first conclusion indicates that the correlation between EEG signals and foot position decreases during the motor imagery (MI) task and increases during the relaxation (relax) task. This observation can be explained by the phenomenon of Event-Related Desynchronization (ERD) and Event-Related Synchronization (ERS) [57,58]. During motor imagery, activity in the alpha and beta bands undergoes desynchronization (ERD) for foot MI [59], reflecting increased cortical activation and the cognitive demand required to mentally simulate movements. This desynchronization introduces greater variability and noise into the EEG signals, thereby reducing the correlation with foot position signals. In contrast, during relaxation periods, synchronization of the alpha and beta rhythms (ERS) is observed, indicating reduced cortical activity and greater coherence in the EEG signals. This synchronization facilitates a higher correlation between EEG signals and foot position due to less interference and variability in the neuronal signal. These findings underscore the importance of cortical state in modulating the correlation between brain signals and motor variables, highlighting the significant impact of cortical rhythms on the interpretation of neurophysiological data.

Another key conclusion, which evaluated which frequency band presents the highest correlation values, aligns with the previous conclusion. The maximum correlation between EEG signals and foot position during FP and FD movements in the 8–20 Hz band (alpha and beta) is due to several key reasons. Alpha activity (8–12 Hz) is associated with states of relaxation and inhibitory processes in the motor cortex, facilitating greater coherence of EEG signals during relaxation. Beta activity (13–20 Hz) is linked to active motor control and sensorimotor integration, which are essential both for the planning and execution of movements during motor imagery [60,61]. Furthermore, event-related desynchronization (ERD) in these bands reflects the cortical activation necessary for motor processing, while event-related synchronization (ERS) during relaxation provides a more stable EEG signal, increasing the correlation with foot position. These characteristics make the alpha and beta bands fundamental for the correlation between EEG signals and motor movements.

In other initial studies aligned with this research, which aimed to discern between motor imagery and motor action using the Stockwell transform [62], it was concluded that the alpha and beta bands provided the most information. This result is consistent with the findings obtained in that study. Another study focusing on classifying FD and FP movements at different levels of movement amplitude [28] obtained a classification percentage of 22.19% using EEG. This result aligns with the low correlation values obtained in this research. Additionally, that study also used fMRI as an acquisition method, achieving a classification percentage of 65.64%. The areas of study with the best results are the same as those worked on in this research. In another similar study that combined EEG and EMG for FD and FP classification, accuracies around 90% were achieved, but without performing any tasks involving cognitive engagement [63].

Aligned with the discussions conducted for correlation analysis, the development of a classification algorithm for motor imagery and relaxation tasks is proposed. In the movement models, the average accuracy values were STFT = 93.84%, HHT = 73.50%, ST = 89.28%, and CT = 91.55%. STFT stands out as the most effective and consistent method, showing high accuracy in classifying motor imagery and relaxation tasks. For static models, the averages were STFT = 91.96%, HHT = 68.97%, ST = 88.42%, and CT = 89.79%. Although the values are slightly lower than those obtained in the movement models, STFT continues to demonstrate its superiority. When comparing these results with those obtained in other studies using time-frequency transforms but with traditional classification algorithms, we see that these results are superior, especially in the case of the Stockwell Transform [17].

## 6. Limitations

It is important to recognize the limitations of this study for a comprehensive understanding of its findings and applicability.

At the signal processing level, time-frequency transforms present the problem of border effects. As mentioned in Section 3.2, this issue has been addressed by extracting the central second of the analysis window. However, this is also counterproductive, as it results in half the analysis data compared to the STFT method, which could potentially affect the effectiveness of these transforms. This limitation is particularly relevant because time-frequency transforms such as HHT, ST and CH tend to be more sensitive to short analysis windows. With less data per epoch, there is a higher risk of losing relevant temporal and spectral patterns, especially in non-stationary signals such as EEG. This effect is evident in the results obtained using the HHT method, which showed a notably lower average accuracy compared to STFT in both motion trials (73.5% vs. 93.8%) and static trials (68.97% vs. 91.96%) (see Table 2). Despite this, it is noteworthy that the results obtained with ST and CH are only slightly lower than those with STFT, indicating a good level of robustness even under reduced data conditions.

Another relevant limitation relates to the sampling frequency of the IMUs, which were originally recorded at 100 Hz. Although the signals were resampled to 250 Hz in post-processing to match the EEG sampling rate and facilitate correlation analysis, it is important to note that this resampling does not introduce any new frequency content above 50 Hz due to the Nyquist limit. Consequently, any information above this frequency is not present in the original IMU data. However, as the analyses in this study focused on the 8–20 Hz frequency band, which is well within the reliable range of the IMU, the original sampling rate was sufficient for the purposes of this research. This limitation has been acknowledged for future studies, particularly for applications that may require richer kinematic information or analysis in higher frequency bands.

In relation to the correlation analysis, another limitation is the type of movement used. In this study, a passive movement of the subject was analyzed, as it was completely controlled by the exoskeleton. It would have been interesting to also analyze the correlation response to an active, voluntary movement by the subject. In addition, the movement was always performed with the same amplitude and speed, and it might have been beneficial to vary these parameters, as seen in other studies [57].

Finally, a higher number of subjects would be desirable. However, six is a considerable number compared to other investigations in the field, which rarely use over ten subjects [64], and the minimum number of subjects to define the behavior of a BMI should be between two and ten subjects [64].

## 7. Conclusions

This study shows that time-frequency transformations are effective in analyzing the correlation between EEG signals and ankle position during plantarflexion and dorsiflexion movements. The 8–20 Hz band showed the highest correlation values. There is a clear dependence on the cognitive state of the subject; the correlation decreases during motor imagery and increases during relaxation. This supports the idea that cognitive involvement modulates sensorimotor rhythms and could serve as a useful marker for personalizing therapy. The classification results for motor imagery were excellent, with the STFT method being the most effective, despite the limitations of the border effects. These positive results may be influenced by the training and prior training and evaluation of each user’s motor imagination in the initial MIQ-3 questionnaire.

The use of wearable inertial sensors combined with brain–machine interfaces (BMIs) opens new possibilities for motor rehabilitation, enabling the development of accessible and adaptive assistive devices. In this study, the integration of EEG and IMUs for controlling a low-cost exoskeleton represents a significant advancement in rehabilitation technology accessibility. However, the exoskeleton design still requires optimization to improve control precision and achieve higher correlation values.

Future studies will explore variations in kinematic and dynamic parameters to assess their impact on EEG–movement correlation, as well as the implementation of online testing to validate the effectiveness of the proposed methods in real and dynamic environments.

## Figures and Tables

**Table 1:** Mean frequency values obtained for each IMF and for each model.

Motion (Hz)	0.59 ± 0.15	6.07 ± 1.04	15.23 ± 2.78	32.20 ± 4.64	64.42 ± 7.83
Static (Hz)	0.57 ± 0.12	5.67 ± 0.51	14.18 ± 1.97	30.26 ± 4.24	63.36 ± 9.29
	0–2 Hz	4–8 Hz	8–20 Hz	25–40 Hz	55–75 Hz

**Table 2:** This table shows the results obtained from the classification of the opened-loop experiments. They are divided according to the two types of trial motion and static. It is also divided according to the four frequency-time analysis methods used.

		Motion			Static		
Transform	Subject	ACC Relax	ACC MI	Average	ACC Relax	ACC MI	Average
Short-Time Fourier Transform	S1	0.90 ± 0.11	0.92 ± 0.06	0.91 ± 0.07	0.79 ± 0.18	0.93 ± 0.07	0.86 ± 0.09
	S2	0.94 ± 0.04	0.94 ± 0.04	0.94 ± 0.02	0.95 ± 0.05	0.96 ± 0.03	0.95 ± 0.02
	S3	0.91 ± 0.08	0.94 ± 0.13	0.92 ± 0.09	0.88 ± 0.08	0.93 ± 0.06	0.90 ± 0.06
	S4	0.97 ± 0.03	0.97 ± 0.03	0.97 ± 0.03	0.97 ± 0.03	0.90 ± 0.05	0.89 ± 0.04
	S5	0.92 ± 0.08	0.94 ± 0.06	0.93 ± 0.06	0.87 ± 0.09	0.90 ± 0.05	0.89 ± 0.04
	S6	0.96 ± 0.03	0.94 ± 0.03	0.96 ± 0.02	0.96 ± 0.05	0.90 ± 0.09	0.95 ± 0.06
Stockwell Transform	S1	0.87 ± 0.12	0.87 ± 0.07	0.87 ± 0.07	0.84 ± 0.13	0.87 ± 0.08	0.85 ± 0.09
	S2	0.92 ± 0.05	0.89 ± 0.05	0.90 ± 0.03	0.91 ± 0.07	0.92 ± 0.05	0.92 ± 0.03
	S3	0.89 ± 0.09	0.94 ± 0.02	0.91 ± 0.05	0.83 ± 0.13	0.91 ± 0.06	0.87 ± 0.08
	S4	0.94 ± 0.03	0.93 ± 0.05	0.93 ± 0.03	0.93 ± 0.04	0.93 ± 0.04	0.93 ± 0.03
	S5	0.73 ± 0.11	0.88 ± 0.09	0.81 ± 0.06	0.85 ± 0.10	0.81 ± 0.08	0.83 ± 0.06
	S6	0.93 ± 0.04	0.88 ± 0.03	0.93 ± 0.02	0.92 ± 0.05	0.81 ± 0.10	0.91 ± 0.06
Hilbert-Huang	S1	0.66 ± 0.14	0.67 ± 0.15	0.66 ± 0.05	0.70 ± 0.07	0.60 ± 0.07	0.65 ± 0.03
	S2	0.79 ± 0.10	0.75 ± 0.08	0.77 ± 0.05	0.77 ± 0.08	0.78 ± 0.10	0.77 ± 0.04
	S3	0.90 ± 0.09	0.95 ± 0.08	0.92 ± 0.08	0.60 ± 0.69	0.74 ± 0.11	0.67 ± 0.04
	S4	0.67 ± 0.10	0.77 ± 0.08	0.72 ± 0.05	0.68 ± 0.11	0.81 ± 0.07	0.75 ± 0.04
	S5	0.65 ± 0.17	0.72 ± 0.13	0.68 ± 0.06	0.57 ± 0.12	0.69 ± 0.12	0.63 ± 0.04
	S6	0.65 ± 0.10	0.72 ± 0.10	0.64 ± 0.04	0.64 ± 0.09	0.69 ± 0.07	0.67 ± 0.05
Chirplet Transform	S1	0.90 ± 0.06	0.88 ± 0.13	0.89 ± 0.08	0.85 ± 0.10	0.90 ± 0.08	0.88 ± 0.07
	S2	0.89 ± 0.04	0.92 ± 0.06	0.90 ± 0.03	0.98 ± 0.04	0.93 ± 0.04	0.95 ± 0.03
	S3	0.90 ± 0.09	0.89 ± 0.12	0.90 ± 0.10	0.88 ± 0.09	0.88 ± 0.08	0.88 ± 0.07
	S4	0.94 ± 0.03	0.96 ± 0.03	0.95 ± 0.02	0.92 ± 0.04	0.97 ± 0.03	0.94 ± 0.03
	S5	0.90 ± 0.07	0.90 ± 0.06	0.90 ± 0.05	0.84 ± 0.13	0.79 ± 0.09	0.82 ± 0.05
	S6	0.96 ± 0.02	0.90 ± 0.04	0.95 ± 0.02	0.94 ± 0.09	0.92 ± 0.09	0.93 ± 0.09

## Data Availability

The dataset supporting the conclusions of this article is available in the Zenodo repository, https://doi.org/10.5281/zenodo.14672334 (accessed on 4 March 2025).

## References

[1] He Y., Eguren D., Azorín J.M., Grossman R.G., Luu T.P., Contreras-Vidal J.L. (2018). Brain-machine interfaces for controlling lower-limb powered robotic systems. J. Neural Eng..

[2] King C.E., Wang P.T., Chui L.A., Do A.H., Nenadic Z. (2013). Operation of a brain-computer interface walking simulator for individuals with spinal cord injury. J. Neuroeng. Rehabil..

[3] Yang H., Guan C., Wang C.C., Ang K.K. (2015). Detection of motor imagery of brisk walking from electroencephalogram. J. Neurosci. Methods.

[4] Ferrero L., Ortiz M., Quiles V., Iáñez E., Flores J.A., Azorín J.M. (2021). Brain symmetry analysis during the use of a BCI based on motor imagery for the control of a lower-limb exoskeleton. Symmetry.

[5] Shyam A.K.B., Wadhwani, Kumar Singh K., Singh S., Srivastava S., Bajpai M.K. (2023). Investigation of the Impact of EEG Signal Processing Techniques on Classification Performance. Proceedings of the Machine Vision and Augmented Intelligence.

[6] Pup F.D., Zanola A., Tshimanga L.F., Bertoldo A., Atzori M. (2025). The More, the Better? Evaluating the Role of EEG Preprocessing for Deep Learning Applications. IEEE Trans. Neural Syst. Rehabil. Eng..

[7] Jiang X., Bian G.B., Tian Z. (2019). Removal of artifacts from EEG signals: A review. Sensors.

[8] Ramoser H., Müller-Gerking J., Pfurtscheller G. (2000). Optimal spatial filtering of single trial EEG during imagined hand movement. IEEE Trans. Rehabil. Eng..

[9] Herman P., Prasad G., McGinnity T.M., Coyle D. (2008). Comparative analysis of spectral approaches to feature extraction for EEG-based motor imagery classification. IEEE Trans. Neural Syst. Rehabil. Eng..

[10] Morales S., Bowers M.E. (2022). Time-frequency analysis methods and their application in developmental EEG data. Dev. Cogn. Neurosci..

[11] Zhang L., Wu D., Zhi L. Method of removing noise from EEG signals based on HHT method. Proceedings of the 2009 1st International Conference on Information Science and Engineering, ICISE 2009.

[12] Jindal K., Upadhyay R., Singh H.S. (2020). Application of hybrid GLCT-PICA de-noising method in automated EEG artifact removal. Biomed. Signal Process. Control.

[13] Bhargava A., Mann S. Adaptive Chirplet Transform-Based Machine Learning for P300 Brainwave Classification. Proceedings of the Proceedings—2020 IEEE EMBS Conference on Biomedical Engineering and Sciences, IECBES 2020.

[14] Jiang Y., Chen W., Li M., Zhang T., You Y. (2021). Synchroextracting chirplet transform-based epileptic seizures detection using EEG. Biomed. Signal Process. Control.

[15] Kaur M., Upadhyay R., Kumar V. (2024). A Hybrid Deep Learning Framework Using Scaling-Basis Chirplet Transform for Motor Imagery EEG Recognition in Brain–Computer Interface Applications. Int. J. Imaging Syst. Technol..

[16] Kalbkhani H., Shayesteh M.G. (2017). Stockwell transform for epileptic seizure detection from EEG signals. Biomed. Signal Process. Control.

[17] Ortiz M., Rodríguez-Ugarte M., Iáñez E., Azorín J.M. (2017). Application of the stockwell transform to electroencephalographic signal analysis during gait cycle. Front. Neurosci..

[18] Ortiz M., Ferrero L., Iáñez E., Azorín J.M., Contreras-Vidal J.L. (2020). Sensory Integration in Human Movement: A New Brain-Machine Interface Based on Gamma Band and Attention Level for Controlling a Lower-Limb Exoskeleton. Front. Bioeng. Biotechnol..

[19] Salimpour S., Kalbkhani H., Seyyedi S., Solouk V. (2022). Stockwell transform and semi-supervised feature selection from deep features for classification of BCI signals. Sci. Rep..

[20] Sethi S., Upadhyay R., Singh H.S. (2018). Stockwell-common spatial pattern technique for motor imagery-based Brain Computer Interface design. Comput. Electr. Eng..

[21] Wang L., Xu G., Wang J., Yang S., Yan W. Application of Hilbert-Huang transform for the study of motor imagery tasks. Proceedings of the 30th Annual International Conference of the IEEE Engineering in Medicine and Biology Society.

[22] Yu Y., Zhao Y., Li S., Shi L., Li Z., Dong B. (2017). Motor imagery EEG discrimination using Hilbert-Huang Entropy. Biomed. Res..

[23] Ortiz M., Iáñez E., Contreras-Vidal J.L., Azorín J.M. (2020). Analysis of the EEG Rhythms Based on the Empirical Mode Decomposition During Motor Imagery When Using a Lower-Limb Exoskeleton. A Case Study. Front. Neurorobot..

[24] Kim H., Yoshimura N., Koike Y. (2019). Characteristics of Kinematic Parameters in Decoding Intended Reaching Movements Using Electroencephalography (EEG). Front. Neurosci..

[25] Lv J., Li Y., Gu Z. (2010). Decoding hand movement velocity from electroencephalogram signals during a drawing task. BioMed Eng. Online.

[26] Robinson N., Guan C., Vinod A.P. (2015). Adaptive estimation of hand movement trajectory in an EEG based brain-computer interface system. J. Neural Eng..

[27] Meier J.D., Aflalo T.N., Kastner S., Graziano M.S. (2008). Complex organization of human primary motor cortex: A high-resolution fMRI study. J. Neurophysiol..

[28] Tobar A.M., Hyoudou R., Kita K., Nakamura T., Kambara H., Ogata Y., Hanakawa T., Koike Y., Yoshimura N. (2018). Decoding of ankle flexion and extension from cortical current sources estimated from non-invasive brain activity recording methods. Front. Neurosci..

[29] O’Keeffe R., Shirazi S.Y., Vecchio A.D., Ibanez J., Mrachacz-Kersting N., Bighamian R., Rizzo J., Farina D., Atashzar S.F. (2022). Low-frequency motor cortex EEG predicts four levels of rate of change of force during ankle dorsiflexion. bioRxiv.

[30] Jiang N., Gizzi L., Mrachacz-Kersting N., Dremstrup K., Farina D. (2015). A brain-computer interface for single-trial detection of gait initiation from movement related cortical potentials. Clin. Neurophysiol..

[31] Donati A.R., Shokur S., Morya E., Campos D.S., Moioli R.C., Gitti C.M., Augusto P.B., Tripodi S., Pires C.G., Pereira G.A. (2016). Long-Term Training with a Brain-Machine Interface-Based Gait Protocol Induces Partial Neurological Recovery in Paraplegic Patients. Sci. Rep..

[32] Pinto D., Garnier M., Barbas J., Chang S.H., Charlifue S., Field-Fote E., Furbish C., Tefertiller C., Mummidisetty C.K., Taylor H. (2020). Budget impact analysis of robotic exoskeleton use for locomotor training following spinal cord injury in four SCI Model Systems. J. Neuroeng. Rehabil..

[33] Collins S.H., Wiggin M.B., Sawicki G.S. (2015). Reducing the energy cost of human walking using an unpowered exoskeleton. Nature.

[34] Trapero-Asenjo S., Gallego-Izquierdo T., Pecos-Martín D., Nunez-Nagy S. (2021). Translation, cultural adaptation, and validation of the Spanish version of the Movement Imagery Questionnaire-3 (MIQ-3). Musculoskelet. Sci. Pract..

[35] g.tec Medical Engineering GmbH (2025). g.Nautilus PRO FLEXIBLE–Wireless EEG/ECG/EMG System. https://www.gtec.at/product/g-nautilus-pro-flexible.

[36] WIT Motion (2025). WT901BLECL BLE 9-Axis High-Precision Attitude Sensor IMU. https://www.wit-motion.com/BLE/52.html.

[37] Ferrero L., Quiles V., Ortiz M., Iáñez E., Azorín J.M. (2021). A BMI based on motor imagery and attention for commanding a lower-limb robotic exoskeleton: A case study. Appl. Sci..

[38] Kilicarslan A., Grossman R.G., Contreras-Vidal J.L. (2016). A robust adaptive denoising framework for real-time artifact removal in scalp EEG measurements. J. Neural Eng..

[39] Goldberg G. (1985). Supplementary motor area structure and function: Review and hypotheses. Behav. Brain Sci..

[40] Boehme T.K., Bracewell R. (1966). The Fourier Transform and its Applications. Am. Math. Mon..

[41] Huang N.E., Shen Z., Long S.R., Wu M.C., Shih H.H., chyuan Yen N., Tung C.C., Liu H.H. (1996). The empirical mode decomposition and the Hilbert spectrum for nonlinear and non-stationary time series analysis. Proc. R. Soc. A.

[42] Stockwell R.G., Mansinha L., Lowe R.P. (1996). Localization of the complex spectrum: The S transform. IEEE Trans. Signal Process..

[43] Mann S., Haykin S. (1995). The Chirplet Transform: Physical Considerations. IEEE Trans. Signal Process..

[44] Fu K., Qu J., Chai Y., Zou T. (2015). Hilbert marginal spectrum analysis for automatic seizure detection in EEG signals. Biomed. Signal Process. Control.

[45] Dragomiretskiy K., Zosso D. (2014). Variational mode decomposition. IEEE Trans. Signal Process..

[46] Peng Z.K., Meng G., Chu F.L., Lang Z.Q., Zhang W.M., Yang Y. (2011). Polynomial chirplet transform with application to instantaneous frequency estimation. IEEE Trans. Instrum. Meas..

[47] Delgado A.L., Delisle-Rodriguez D., da Rocha A.F., Figueroa E.S., López-Delis A. (2024). Revisión sobre nuevos enfoques de terapias de neurorrehabilitación para pacientes con trastornos neurológicos mediante dispositivos de pedaleo. Neurol. Argent..

[48] Sugumar D., Vanathi P.T. (2017). EEG Signal Separation using Improved EEMD-Fast IVA Algorithm. Asian J. Res. Soc. Sci. Humanit..

[49] Field A. (2013). Discovering Statistics Using SPSS Statistics.

[50] Box G.E.P., Jenkins G.M., Reinsel G.C. (1994). Time Series Analysis: Forecasting and Control.

[51] Lawhern V.J., Solon A.J., Waytowich N.R., Gordon S.M., Hung C.P., Lance B.J. (2018). EEGNet: A compact convolutional neural network for EEG-based brain-computer interfaces. J. Neural Eng..

[52] Loshchilov I., Hutter F. SGDR: Stochastic gradient descent with warm restarts. Proceedings of the 5th International Conference on Learning Representations, ICLR 2017-Conference Track Proceedings.

[53] Pfurtscheller G., Neuper C. (1997). Motor imagery activates primary sensorimotor area in humans. Neurosci. Lett..

[54] Friedman M. (1937). The use of ranks to avoid the assumption of normality implicit in the analysis of variance. J. Am. Stat. Assoc..

[55] Wilcoxon F. (1945). Individual comparisons by ranking methods. Biometrics.

[56] Kruskal W.H., Wallis W.A. (1952). Use of ranks in one-criterion variance analysis. J. Am. Stat. Assoc..

[57] Jeon Y., Nam C.S., Kim Y.J., Whang M.C. (2011). Event-related (De)synchronization (ERD/ERS) during motor imagery tasks: Implications for brain-computer interfaces. Int. J. Ind. Ergon..

[58] Nakayashiki K., Saeki M., Takata Y., Hayashi Y., Kondo T. (2014). Modulation of event-related desynchronization during kinematic and kinetic hand movements. J. Neuroeng. Rehabil..

[59] Pfurtscheller G., Brunner C., Schlögl A., da Silva F.H.L. (2006). Mu rhythm (de)synchronization and EEG single-trial classification of different motor imagery tasks. NeuroImage.

[60] Severens M., Nienhuis B., Desain P., Duysens J. Feasibility of measuring event Related Desynchronization with electroencephalography during walking. Proceedings of the Annual International Conference of the IEEE Engineering in Medicine and Biology Society, EMBS.

[61] Wagner J., Solis-Escalante T., Grieshofer P., Neuper C., Müller-Putz G., Scherer R. (2012). Level of participation in robotic-assisted treadmill walking modulates midline sensorimotor EEG rhythms in able-bodied subjects. NeuroImage.

[62] Polo-Hortigüela C., Juan J.V., Ortiz M., Iañez E., Azorín J.M. Análisis de señales EEG en movimientos de flexión plantar y dorsal mediante el empleo de un exoesqueleto de bajo coste para la caracterización de la acción motora. Proceedings of the XLI Congreso Anual de la Sociedad Española de Ingeniería Biomédica.

[63] Hooda N., Das R., Kumar N. (2020). Fusion of EEG and EMG signals for classification of unilateral foot movements. Biomed. Signal Process. Control.

[64] Wierzgała P., Zapała D., Wojcik G.M., Masiak J. (2018). Most popular signal processing methods in motor-imagery BCI: A review and meta-analysis. Front. Neuroinform..

